# Prediction of Serious Adverse Events Associated With Pediatric Cardiac Catheterizations: External Model Validation of the CRISP Scoring Method

**DOI:** 10.1016/j.jscai.2025.104155

**Published:** 2026-02-03

**Authors:** Yoshihiko Kurita, Rajiv Chaturvedi, Lee Benson

**Affiliations:** a Department of Pediatrics, Kochi Health Sciences Center, Kochi, Japan; b Department of Pediatrics, Division of Cardiology, The Labatt Family Heart Center, The Hospital for Sick Children, The Temerty School of Medicine, University of Toronto, Toronto, Canada

**Keywords:** Catheterization Risk Score for Pediatrics, external validation, pediatric cardiac catheterization, risk stratification, serious adverse event

## Abstract

**Background:**

A methodology for predicting pediatric cardiac catheterization-related complications can assist interventionists in planning treatment strategies, obtaining informed consent, and allocating medical resources. We aimed to perform an external validation of the Catheterization Risk Score for Pediatrics (CRISP).

**Methods:**

We retrospectively reviewed 3093 consecutive catheterizations performed between January 2016 and March 2021 to calculate the CRISP. Model performance was assessed using discrimination (area under the receiver operating characteristic curve [AUC]) and calibration (observed and predicted risk).

**Results:**

Serious adverse events (SAE) occurred in 200 procedures (6.5%, 208 events). The most frequent complication was arterial occlusive thrombosis at the access site (1.8%). Nine catheterization-related deaths were identified. The mean CRISP score was 5.74 ± 3.43. SAE incidence increased across categories, ranging from 1.5% in category 1 to 36.4% in category 5. The CRISP score and categories demonstrated good discriminative power (AUC, 0.729; 95% CI, 0.695-0.764; AUC, 0.717, 95% CI, 0.682-0.752).

**Conclusions:**

The CRISP scoring method demonstrated good predictive performance in identifying SAE. This external validation supports procedural planning and may improve communication with patients and families, thereby enhancing safety and quality of care.

## Introduction

Quality assurance improvements in pediatric cardiac catheterization have had an increased focus over the last decades with the advent and implementation of interventional procedures. The 2 major domains that have come under scrutiny are efforts to reduce radiation exposure and catheter-related complications.^1^^,^^2^ With the marked increase in catheter procedure complexity, both for diagnostic and interventional formats, there have been efforts to identify those procedures particularly at risk. Accordingly, the incidence of complications associated with pediatric cardiac catheterization has been reported by several institutions since the 1990s.^3^, ^4^, ^5^, ^6^, ^7^, ^8^, ^9^ However, accurate risk assessment from these studies was difficult as they were single-center retrospective reviews, with varying complication definitions, diverse patient backgrounds and procedures, varying operator experience, relatively low event rates and small sample sizes. In an attempt to address this, several large-scale procedural registries were established to create a risk assessment profile for various pediatric cardiac catheterization procedures.^10^, ^11^, ^12^, ^13^, ^14^, ^15^, ^16^, ^17^ The Catheterization Risk Score for Pediatrics (CRISP) model, which used patient risk stratification to predict complications based on data obtained before the catheter procedure, was developed from the Congenital Cardiovascular Interventional Study Consortium (CCISC) registry, and more recently, a revised CRISP model was introduced.^18^^,^^19^ The original CRISP model demonstrated promising internal validity, primarily evaluated through the area under the receiver operating characteristic curve (AUC) and effectively stratified patients by procedural risk. However, its external generalizability and clinical utility in other institutions with differing case mixes and surveillance standards remain unclear.

The purpose of this study was to externally validate the original CRISP model in our institutional cohort, focusing on its discrimination and calibration performance.

## Materials and methods

### Study design

This is a retrospective observational study. Children aged ≤18 years at the time of cardiac catheterization undergoing diagnostic, interventional or hybrid procedures between January 1, 2016, and March 31, 2021, were reviewed. Exclusion criteria were as follows: children aged ≥18 years; those having undergone a coronary intervention, electrophysiological testing, ablation or pacemaker-ICD therapy; and cases where there was a lack or loss of data, where the score calculations could not be performed. In this study, the definition of variables and the calculation followed those of Nykanen et al.^18^ The CRISP scores range from 0 to 21 points and are divided into 5 risk categories: minimal (0-2), low (3-5), moderate (6-9), high (10-14), and very high (≥15). Supplemental Table S1 shows the point assignment of the CRISP variables. Institutional ethics board approval was waived, as this was a retrospective review and was undertaken as a quality improvement project.

### Data collection and CRISP score calculation

We reviewed our complications database to collect information regarding the children's demographic characteristics, physical conditions, procedural characteristics, and adverse events (AE). Specifically, we collected the following clinical information: age, body weight, any inotropic supports, associated organ failure, therapeutic support, oxygen saturation, hemodynamic and morphological characteristics, cardiac diagnosis, and the procedures during the catheterizations (diagnostic, interventional, or hybrid).

The CRISP score was calculated using only preprocedural clinical variables as specified in the original model. No postprocedural or outcome-related information was used in the score calculation, thereby maintaining fidelity to the score’s intended use in prospective clinical settings.

### Serious AE definition and evaluation period

The primary outcome was the occurrence of a serious adverse event (SAE). AE were characterized as any anticipated or unanticipated event occurring potentially or definitely as a consequence of performing the catheterization. We identified all AE from our complications database and categorized them in accordance with the original CRISP study and the C3PO registry,^12^ including moderate, major, and catastrophic events. Minor events without clinical consequence were excluded. AE were adjudicated independently by a multidisciplinary review team including a pediatric cardiologist, intensivist, and interventional staff. The evaluation period extended from the time of anesthetic induction through the entirety of the index hospitalization and included catheterization-related SAE that became apparent within 3 months following discharge. This extended period was chosen to capture delayed complications directly attributable to the cardiac catheterization procedure.

### Statistical method and data analysis

Data are presented as frequency (%) for categorical variables and median (IQR) or mean ± SD for continuous variables. Comparison between the 2 groups was done using χ^2^ tests for categorical variables and *t* tests for continuous variables. The discriminative power of the CRISP score was assessed by the AUC. A model whose predictions are 100% wrong has an AUC of 0.0; one whose predictions are 100% correct has an AUC of 1.0. The calibration, which measures the alignment between predicted and observed risks, was assessed by comparing the observed and predicted number of events in the CRISP risk categories. The predicted SAE risks for the CRISP model were based on the suggested risk for the 5 risk categories in the original CRISP development publication. The χ^2^ test was used to assess goodness-of-fit using the 5 CRISP categories (CRISP score 0-2, 3-5, 6-9, 10-14, 15-21). Subgroup analyses were not performed in this study, as the primary objective was to evaluate the overall external validity of the CRISP score in a single-center cohort.

All data were analyzed using SPSS 25 for Windows (IBM Corp), and *P* < .05 was considered to be statistically significant.

## Results

### Baseline demographic characteristics and predictor variables of our validation cohort

In total, there were 3139 catheterizations retrieved from the database. Forty-six cases (1.5% of total cases) were excluded: patient age ≥18 years (n = 18), those having undergone a coronary intervention (n = 2), and electrophysiological procedure (n = 26). A total of 3093procedures were performed on 2104 children: 1023 were female (49%). Table 1 lists the variables from the validation cohort and the original CRISP score development cohort. The median age of the study cohort was 2.89 (0.41-7.75) years, with 11.3% aged <30 days and 63.9% aged >1 year. The median body weight was 12.8 (5.75-24.6) kg, with 3.9% <2.5 kg and 79% >5 kg. There were 811 diagnostic cases (26.2%), 2253 interventional cases (72.8%), and 30 (1.0%) hybrid procedures. General anesthesia was used in 2987 cases (97%). The distribution of procedures was similar over the years reviewed.

**Table 1:** Patient and procedural characteristics of the derivation cohort and the validation cohort.

Characteristics	Original CRISP cohort		Our validation cohort	
	%	SAE (%)	%	SAE (%)
Age				
>1 y	71.0%	3.0%	63.9%	3.5%
30 d to 1 y	22.0%	7.0%	24.8%	10.8%
<30 d	7.0%	12.0%	11.3%	13.5%
Body weight				
>5 kg	86.0%	3.0%	79.3%	4.5%
2.5-5 kg	13.0%	11.0%	16.8%	14.6%
<2.5 kg	1.0%	12.0%	3.9%	12.4%
Inotropic support				
None	93.0%	4.0%	90.7%	6.0%
Yes—stable	6.0%	8.0%	7.3%	8.8%
Yes—unstable/ECMO	1.0%	21.0%	1.9%	18.3%
Systemic illness/organ failure				
None	70.0%	4.0%	73.7%	6.0%
Medically controlled or 1 organ failure	26.0%	5.0%	22.2%	7.1%
Uncontrolled or >1 organ failure	4.0%	13.0%	4.1%	11.0%
Physiologic category				
Category 1	67.0%	3.0%	62.4%	4.3%
Category 2	23.0%	5.0%	26.3%	8.4%
Category 3	10.0%	12.0%	11.3%	14.0%
Pre-cath diagnosis				
Category 1	50.0%	3.0%	45.3%	3.7%
Category 2	46.0%	6.0%	51.6%	8.6%
Category 3	4.0%	5.0%	3.1%	11.5%
Procedure risk category				
Category 1	86.0%	4.0%	71.1%	4.3%
Category 2	12.0%	8.0%	22.7%	9.7%
Category 3	2.0%	15.0%	6.3%	19.2%
Procedure type				
Diagnostic	38.0%	3.0%	26.2%	4.8%
Interventional	61.0%	5.0%	72.8%	6.9%
Hybrid	1.0%	12.0%	1.0%	20.0%

CRISP, Catheterization Risk Score for Pediatrics; ECMO, extracorporeal membrane oxygenation; SAE, serious adverse event.

### SAE

A total of 2674 (86.4%) were performed in patients with no complications. Two hundred catheterizations (6.5%) were performed in patients who experienced 208 SAE: moderate 128 (4.1%), major 58 (1.9%), or catastrophic 14 (0.5%). Table 2 summarizes the SAE details. Eight procedures were associated with more than 1 complication. The most common complication was arterial compromise, with an occlusive thrombosis in 55 procedures (1.78%). Eleven cases (0.4%) required extracorporeal membrane oxygenation cannulation during the procedure. Eight children (0.3%) died related to the complication. Three children were readmitted to the hospital after being discharged due to a catheterization-related complication, which included an access site abscess, a retroperitoneal hematoma and severe hypotension and desaturation.

**Table 2:** Reported SAE in our validation cohort.

Complication detail	No. of SAE (% of all catheterizations)
Occlusive arterial thrombosis of the access site	55 (1.78)
Hemodynamic instability requiring inotrope/resuscitation	31 (1.00)
Vascular injury	27 (0.87)
Hemodynamically unstable arrhythmias	19 (0.61)
Device/wire issues	18 (0.58)
Pulmonary hemorrhage/hemothorax	11 (0.36)
Complete atrioventricular block	11 (0.36)
Cardiac perforation/pericardial effusion requiring intervention	6 (0.19)
Formation of an aneurysm/fistula/abscess of the access site	5 (0.16)
Occlusive venous thrombosis of the access site	4 (0.13)
Coronary artery issues	3 (0.10)
Issues related to the induction of anesthesia	3 (0.10)
Postextubation respiratory distress	3 (0.10)
Systemic thromboembolic events	3 (0.10)
Ischemic limb	2 (0.06)
Retroperitoneal bleeding/hematoma	2 (0.06)
Serious bleeding due to vascular injury	2 (0.06)
Cerebral infarction	2 (0.06)
Significantly worsened valve regurgitation	1 (0.03)

SAE, serious adverse event.

### Deaths

Eight children died due to a catheter-related complication. The median age at death was 3.6 years (1.8 months to 8.1 years), and the median weight was 11.2 kg (1.7 kg to 48.2 kg). Half of the children were aged >1 year. Five children underwent an interventional catheterization, and 3 a diagnostic study. Two children died during the catheterization, and 6 in the intensive care unit after the procedure. The causes of death were cardiac arrest, low cardiac output syndrome, severe hypoxia, ischemic heart failure, and pulmonary artery hypertension. In addition, there were 3 deaths during hospitalization after catheterization, which were excluded as an SAE because their clinical status was very poor prior to the cardiac catheterization, and the procedure was not considered to be the direct cause of death.

### Validation of CRISP score and risk category

**Figure 1:**
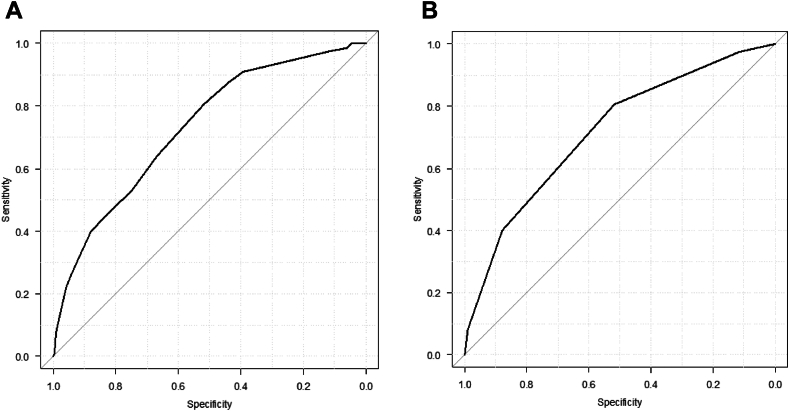
**Area under the receiver operating characteristic (AUC) curve of the Catheterization Risk Score for Pediatrics (CRISP) score and CRISP risk category in our validation cohort.** (A) AUC for CRISP score (0-21): AUC is 0.729 (95% CI, 0.695-0.764). (B) AUC for CRISP category (1-5): AUC is 0.717 (95% CI, 0.682-0.752).

**Figure 2:**
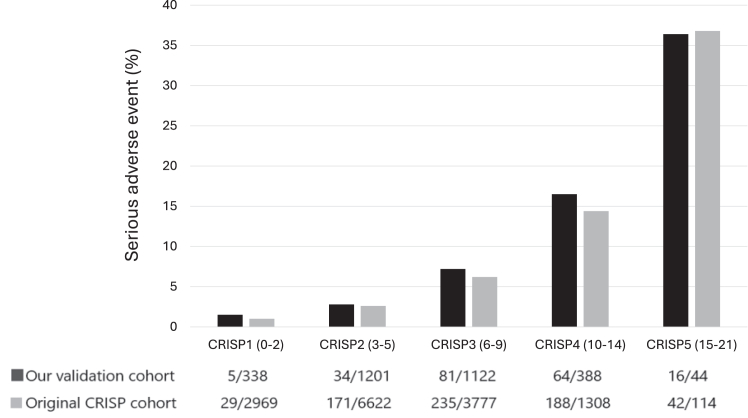
**Increasing risk of serious adverse events with increasing Catheterization Risk Score for Pediatrics (CRISP) category among both cohorts.***P* < .0001 by the Cochran-Armitage test.

The distribution of the CRISP scores (0-21) is shown in Supplemental Table S2. There were no patients with CRISP scores of 20 or 21, and no SAE occurred in patients with scores of 0 or 19. The mean CRISP score was 4.72 ± 3.25 and 5.74 ± 3.43 (*P* < .001) in the original CRISP cohort and our cohort, respectively. The number of cases and SAE and the probability of an SAE occurrence according to CRISP categories (1 to 5) are shown in Table 3.^18^ The most common risk group was CRISP 2 (1201 cases), and the least common was CRISP 5 (44 cases). The incidence rate of SAE increased from 1.5% in CRISP 1 to 36.4% in CRISP 5. The discrepancy between observed to predicted percentage differences ranged from –0.4% to +2.1%. The *P* value of the χ^2^ test in our cohort was 0.35. The AUC for the CRISP scores (0-21) and risk categories (1-5) are shown in Figure 1. The C-index was 0.729 (95% CI, 0.695-0.764) and 0.717 (95% CI, 0.682-0.752), in the CRISP original cohort and our cohorts, respectively. The comparison between these 2 cohorts according to CRISP risk categories is shown in Figure 2. The incidence of SAE increased consistently with higher CRISP categories, demonstrating good concordance with the original CRISP cohort. Although the observed incidence in category 5 was slightly lower than predicted, the overall trend of rising risk with higher categories was maintained.

**Table 3:** Observed vs predicted % SAE by CRISP risk category.

CRISP category (CRISP score)	N	No. of SAE	Observed % SAE	Predicted % SAE^a^	Difference % SAE^b^
CRISP 1 (0-2)	338	5	1.5%	1.0%	+0.5%
CRISP 2 (3-5)	1201	34	2.8%	2.6%	+0.2%
CRISP 3 (6-9)	1122	81	7.2%	6.2%	+1.0%
CRISP 4 (10-14)	388	64	16.5%	14.4%	+2.1%
CRISP 5 (15-21)	44	16	36.4%	36.8%	–0.4%
Total	3093	200	6.5%	4.5%	

χ^2^ and *P* values are 4.09 and 0.35, respectively.

CRISP, Catheterization Risk Score for Pediatrics; SAE, serious adverse event.

a Predicted % SAE from the original CRISP article, published by Nykanen et al.^18^

b Difference % SAE; observed % – predicted %.

## Discussion

Given the increasing procedural complexity of pediatric cardiac catheterization, the development of reliable complication risk prediction models has received appropriate attention. The Congenital Heart Disease Adjustment for Risk Method and the Improving Pediatric and Adult Congenital Treatment (IMPACT) models were developed from the C3PO and IMPACT registries, respectively.^14^^,^^15^ The Congenital Heart Disease Adjustment for Risk Method model, reported by Bergersen et al,^15^ classified AE severity into 5 grades and assessed the incidence of moderate or greater AE according to treatment and hemodynamic parameters. The IMPACT model used patient characteristics in addition to procedure and hemodynamic parameters for assessing complications. However, the CRISP score was developed as a tool for preprocedural evaluation. It is important to distinguish between risk prediction and risk adjustment. Risk prediction models aim to estimate an individual patient’s likelihood of experiencing an adverse outcome before the procedure, thereby informing clinical decision-making, such as procedural planning or resource allocation. In contrast, risk adjustment is used retrospectively for benchmarking and comparing outcomes across institutions or time periods. The CRISP score is intended for prospective risk prediction, not for institutional comparison or quality metrics, and should be interpreted as such. In this regard, there have been limited studies using the CRISP score in evaluating procedural risk.^20^, ^21^, ^22^ In this study, we performed an external validation of the CRISP score using an external cohort outside of the CCISC registry.

The incidence of SAE in our cohort was 6.5%, which is higher than that in the original CRISP development cohort (4.5%). Previous studies have reported that the incidence of SAE in pediatric cardiac catheterizations ranges from 3.4% to 12%.^3^, ^4^, ^5^^,^^7^, ^8^, ^9^^,^^12^^,^^14^^,^^15^^,^^23^ This variability is in part due to differences in case mix (interventional or diagnostic), patient ages, and in the definition of complication and type and degree of surveillance. From the C3PO and IMPACT reports, the incidence of a SAE was approximately 6.2% and 7.1%, respectively, similar to that obtained in our cohort, slightly less than catheterization-related complications published by our institution in 1998 and 2008, which were around 8.0%.^6^^,^^23^ Taking into account that the definition of complications and severity differs among institutions and registries, the incidence of complications in our cohort is compatible with other reports. The SAE complication rate in the original CRISP article was 4.5%, with a difference of approximately 2% from our validation cohort. Because the mean CRISP score in our cohort was found to be significantly higher than the original CRISP cohort, it is possible that the higher risk profile of this validation cohort may have contributed to the difference in the incidence of complications. The incidence of occlusive arterial thrombosis at the vascular access site was 1.8% in our cohort, but reported to be 0.7% in the CRISP development cohort. In our cohort, a relatively high proportion of SAE were related to arterial occlusive thrombosis. One possible explanation for this discrepancy compared to the original CRISP cohort is the difference in event surveillance and recognition. The original CRISP model was developed using a multicenter registry (CCISC), in which variations in data collection and event adjudication across institutions might have led to underreporting of certain complications, such as access site thrombosis. In contrast, our single-center cohort maintained a high level of vigilance for vascular access complications, with systematic monitoring and documentation protocols, which likely contributed to a higher detection rate of arterial thromboembolism. Therefore, the observed difference may not reflect a true increase in event incidence but rather a difference in ascertainment intensity. Previous reports from other institutions have documented that the incidence of arterial occlusive thromboembolism ranges from 0.6% to 9.6%,^2^^,^^6^^,^^12^^,^^24^, ^25^, ^26^, ^27^ suggesting that the incidence in our cohort was representative.

The calculated C-index was 0.729 for CRISP scores (0-21) and 0.717 for the CRISP categories.^1^, ^2^, ^3^, ^4^, ^5^ The predictive performance in our validation cohort tended to be lower than that in the CRISP derivation cohort; as such, the C-index of our cohort was lower than that in the original CRISP development cohort. Nevertheless, both the scores and categories have moderate to good discriminative power in our cohort. Although AUC is a useful summary measure of discrimination, it does not fully capture clinical utility. Additional metrics such as net reclassification improvement or decision curve analysis could offer further insights into the score’s impact on clinical decision-making. However, these were beyond the scope of the present study, which aimed solely to validate the CRISP score. When each category in the original CRISP development cohort and our validation cohort was investigated, the probability of an SAE in our cohort was found to be slightly higher than the original development cohort in all categories except category 5, with the χ^2^ test implying a better model fit. On the whole, the CRISP categories have a good correlation in our validation cohort. We also noted a positive correlation: higher CRSP categories were associated with higher incidences of SAE. This finding indicates that the CRISP categories can accurately distinguish between high- and low-risk catheterizations. In practice, the score can be shared with all staff laboratory personnel involved in the procedure and also with the patients and their families, providing additional information in the informed consent process. For example, the ability to discriminate between high- and low-risk catheterizations may assist in health care resource allocation decisions, eg, preparing extracorporeal membrane oxygenation support in advance.

The CRISP scoring method is simple to calculate. However, this metric has problems regarding some predictive variables. For example, to obtain a physiological score at the time of score calculation, values of the right ventricular pressure and pulmonary vascular resistance are required. However, because the score is a preprocedural scoring system, such values must be estimated from echocardiography, cardiac magnetic resonance imaging, or previous cardiac catheterization. Thus, high-quality and reliable precatheterization cross-sectional image evaluation is necessary for better predictive performance. Additionally, an evaluation of detailed physical findings is essential. This issue was pointed out by Hill et al,^19^ and the CRISP score model was refined and revised. Although the revised CRISP score has a slightly lower overall predictive power than that of the original CRISP score, it continues to perform well as a prediction model applicable in clinical settings. The revised CRISP score was not evaluated in our cohort, and other predictive factors and modifiers were also not assessed; these will be addressed in future studies.

The overall study design and the relationship between CRISP categories and SAE incidence are shown in the Central Illustration.

**Central Illustration:**
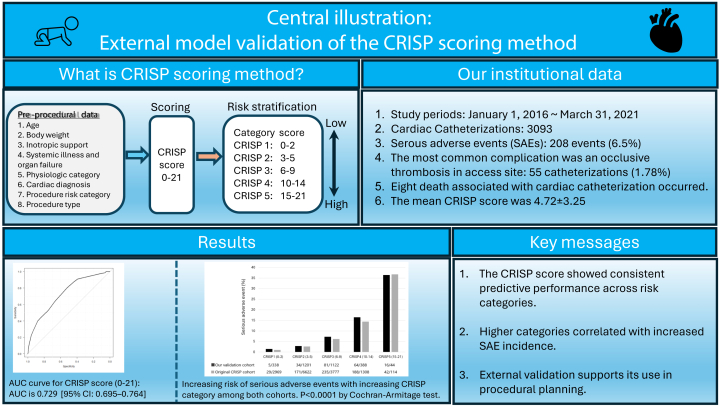
Summary of the study design and external validation of the Catheterization Risk Score for Pediatrics (CRISP) score. Increasing CRISP categories were associated with higher rates of serious adverse events (SAE), confirming consistent predictive performance and supporting its clinical use for risk stratification and procedural planning.

### Limitations

This study has several limitations. First, it was conducted at a single-center with its own case mix and clinical practices, which may limit generalizability to other institutions. Second, the analysis was retrospective, although the CRISP score was calculated using only preprocedural data to preserve its intended prospective use. Third, although AUC and calibration metrics were used to evaluate the score, other measures of clinical utility, such as reclassification or decision analysis, were not assessed. Finally, differences in SAE detection practices, particularly regarding arterial occlusive thrombosis, may have influenced event rates compared to those in the original cohort.

## Conclusion

The CRISP score is a valid tool for preprocedural risk prediction in pediatric cardiac catheterizations. It demonstrates acceptable discrimination and calibration in our external cohort. Although further studies are needed to explore its role in clinical decision-making, our findings support its utility in procedural planning and team communication.
