# Supplementary material for: Prediction of Serious Adverse Events Associated With Pediatric Cardiac Catheterizations: External Model Validation of the CRISP Scoring Method
Source: J Soc Cardiovasc Angiogr Interv. 2026 Feb 3;5(3):104155. doi: 10.1016/j.jscai.2025.104155 (PMC13005389; doi:10.1016/j.jscai.2025.104155)
Supplement: Supplementary Tables S1 and S2 [file mmc1.docx]

Supplemental Table S1

| **Patient Characteristics** | **Points assigned** |
| --- | --- |
| **Age** |  |
| > 1 year | 0 |
| 30days - 1 year | 2 |
| < 30 days | 2 |
| **Weight** |  |
| > 5 kg | 0 |
| 2.5 - 5 kg | 2 |
| < 2.5 kg | 2 |
| **Inotoropic support** |  |
| None or Yes-Stable | 0 |
| Yes-Unstable/ECMO | 2 |
| **Systemic illness/organ failure** |  |
| None or Medically controlled | 0 |
| Uncontrolled/> 1 organ failure | 3 |
| **Physiologic Category** |  |
| Category 1 | 0 |
| Category 2 | 1 |
| Category 3 | 4 |
| **Pre-Cath Diagnosis** |  |
| Category 1 | 0 |
| Category 2 | 2 |
| Category 3 | 2 |
| **Procedure Category** |  |
| Category 1 | 0 |
| Category 2 | 1 |
| Category 3 | 3 |
| **Procedure type** |  |
| Category 1 | 0 |
| Category 2 | 3 |
| Category 3 | 3 |

This table was recreated by the authors based on CRISP scoring system described in Nykanen et al., Catheter Cardiovasc Interv. 2016[18].

It does not reproduce the original published table and is provided solely for clarification of scoring variables.

Supplemental Table S2

Incidence of SAEs by CRISP score in our validation cohort

| CRISP score | Procedure | SAEs | Observed% |
| --- | --- | --- | --- |
| 0 | 126 | 0 | 0.0% |
| 1 | 58 | 3 | 5.2% |
| 2 | 154 | 2 | 1.3% |
| 3 | 818 | 13 | 1.6% |
| 4 | 136 | 7 | 5.1% |
| 5 | 247 | 14 | 5.7% |
| 6 | 466 | 33 | 7.1% |
| 7 | 251 | 22 | 8.8% |
| 8 | 241 | 15 | 6.2% |
| 9 | 164 | 11 | 6.7% |
| 10 | 119 | 15 | 12.6% |
| 11 | 97 | 13 | 13.4% |
| 12 | 52 | 8 | 15.4% |
| 13 | 103 | 24 | 23.3% |
| 14 | 17 | 4 | 23.5% |
| 15 | 7 | 3 | 42.9% |
| 16 | 23 | 11 | 47.8% |
| 17 | 4 | 1 | 25.0% |
| 18 | 6 | 1 | 16.7% |
| 19 | 4 | 0 | 0.0% |
| 20 | 0 | 0 |  |
| 21 | 0 | 0 |  |
| Total | 3093 | 200 | 6.5% |

SAE: Serious Adverse Event

CRISP: Catheterization RISk Score for Pediatrics
